# PEDOT-Doped Mesoporous Nanocarbon Electrodes for High Capacitive Aqueous Symmetric Supercapacitors

**DOI:** 10.3390/nano14141222

**Published:** 2024-07-18

**Authors:** Mohsina Taj, Vinay S. Bhat, Ganesan Sriram, Mahaveer Kurkuri, S. R. Manohara, Paola De Padova, Gurumurthy Hegde

**Affiliations:** 1 Nano-Composites and Materials Research Laboratory, Department of Physics, Siddaganga Institute of Technology (Affiliated to Visvesvaraya Technological University, Belagavi), Tumakuru 572103, Karnataka, India; mohsina.ph@hkbk.edu.in; 2 Department of Physics, HKBK College of Engineering, Bengaluru 560045, Karnataka, India; 3 Department of Materials Science, Mangalore University, Mangalagangotri 574199, Karnataka, India; vinaysb@bmsce.ac.in; 4 School of Chemical Engineering, Yeungnam University, Gyeongsan 38541, Republic of Korea; sriramyu@yu.ac.kr; 5 Centre for Research in Functional Materials (CRFM), Jain (Deemed–to–be University), Jain Global Campus, Bengaluru 562112, Karnataka, India; mahaveer.kurkuri@jainuniversity.ac.in; 6 Consiglio Nazionale delle Ricerche—Istituto di Struttura della Materia (CNR—ISM), Via Fosso del Cavaliere, 100, 00133 Rome, Italy; 7 Istituto Nazionale di Fisica Nucleare—Laboratori Nazionali di Frascati (INFN—LNF), Via E. Fermi, 54, 00040 Frascati, Italy; 8 Centre for Advanced Research and Development, CHRIST (Deemed to be University), Bengaluru 560029, Karnataka, India; 9 Department of Chemistry, CHRIST (Deemed to be University), Bengaluru 560029, Karnataka, India

**Keywords:** carbon nanoparticles, poly(3,4-ethylenedioxythiophene), carbon matrix, mesopores, supercapacitors, nanocomposites, electrode fabrication, onion peels pyrolysis, FESEM/ATR-FTIR/XRD/BET/BJH/CV/GCD/EIS

## Abstract

Poly(3,4-ethylenedioxythiophene) (PEDOT) and PEDOT-functionalized carbon nanoparticles (f-CNPs) were synthesized by in situ chemical oxidative polymerization and pyrolysis methods. f-CNP-PEDOT nanocomposites were prepared by varying the concentration of PEDOT from 1 to 20% by weight (i.e., 1, 2.5, 5, 10, and 20 wt%). Several characterization techniques, such as field-emission scanning electron microscopy (FESEM), attenuated total reflectance-Fourier transform infrared (ATR-FTIR), X-ray diffraction (XRD), N_2_ Brunauer–Emmett–Teller (BET) and Barrett–Joyner–Halenda (BJH) analyses, as well as cyclic voltammetry (CV), galvanostatic charge discharge (GCD), and electrochemical impedance spectroscopy (EIS), were applied to investigate the morphology, the crystalline structure, the N_2_ adsorption/desorption capability, as well as the electrochemical properties of these new synthesized nanocomposite materials. FESEM analysis showed that these nanocomposites have defined porous structures, and BET surface area analysis showed that the standalone f-CNP exhibited the largest surface area of 801.6 m^2^/g, whereas the f-CNP-PEDOT with 20 wt% exhibited the smallest surface area of 116 m^2^/g. The BJH method showed that the nanocomposites were predominantly mesoporous. CV, GCD, and EIS measurements showed that f-CNP functionalized with 5 wt% PEDOT had a higher capacitive performance compared to the individual f-CNPs and PEDOT constituents, exhibiting an extraordinary specific capacitance of 258.7 F/g, at a current density of 0.25 A/g, due to the combined advantage of enhanced electrochemical activity induced by PEDOT doping, and highly developed porosity of f-CNPs. Symmetric aqueous supercapacitor devices were fabricated using the optimized f-CNP-PEDOT doped with 5 wt% of PEDOT as active material, exhibiting a high capacitance of 96.7 F/g at 1.4 V, holding practically their full charge, after 10,000 charge–discharge cycles at 2 A/g, thus providing the highest electrical electrodes performance. Hereafter, this work paves the way for the potential use of f-CNP-PEDOT nanocomposites in the development of high-energy-density supercapacitors.

## 1. Introduction

Supercapacitors are considered promising components of next-generation energy storage devices. They find applications, to cite just a few, in portable electronic devices, telecommunications, as well as uninterrupted power supply, due to their rapid charge–discharge rate, high power density and long cycling stability [1,2,3]. Supercapacitors combine the advantage of the high energy storage capacity of conventional batteries with the greater power delivery capability of traditional capacitors. Based on the charge storing mechanism, the supercapacitors are classified into two types, namely (i) electrochemical double layer capacitors (EDLC) which are based on the accumulation of electrostatic charge at the electrode-electrolyte interface, and (ii) pseudocapacitors, where the energy is stored through reversible redox (Faradaic) reactions at the electrode/electrolyte interface. EDLCs provide greater power density via rapid exchange of absorbed electrolyte ions but exhibit poor energy density. On the contrary, pseudocapacitors provide greater energy density with the exchange of electrons in rapid redox steps. However, due to their poor electrical conductivity, they exhibit low power density and cycling stability [4,5].

Carbon-based nanomaterials and conducting polymers are the most important type of supercapacitor materials [6,7,8,9,10], thanks to the dual important quality of being low cost and exhibiting very high supercapacitor properties. The appropriate combination of these composite compounds can result in a design of novel conceptual electrode materials with increased electrochemical performance [2,11]. The conducting polymers possess functional groups, which have intrinsic pseudocapacitance properties. Among them, poly(3,4-ehylenedioxythiophene) (PEDOT) is a superior supercapacitor material in terms of its processability and electrochemical characteristics [12,13]. PEDOT is an excellent environmentally stable material with rapid charge discharge properties and a narrow band gap. However, its poor life cycle limits its application in supercapacitors [14,15]. The preparation of hybrid electrode materials, by combining PEDOT and carbon materials is an attractive way to overcome this problem.

Carbon nanoparticles, as a new member of the carbon family, have gained substantial attention due to their physicochemical properties, nontoxicity, greater surface area, high electrical conductivity, biocompatibility, and excellent electrochemical activity, which make them potential candidates for the fabrication of supercapacitors, batteries and sensors [16,17]. A blending of functionalized carbon nanoparticles (f-CNPs) and PEDOT was expected to give an excellent supercapacitor material. Therefore, in the present work, a new f-CNP-PEDOT-doped nanocomposites have been developed and their supercapacitor application studied. Highly porous f-CNPs acted as a matrix to be doped by PEDOT polymers, providing a new idea for the design of a completely novel class of materials, well suited in the field of energy storage applications.

The work is organized in four main sections: Introduction, Materials and Methods, Results and Discussion, and Conclusions, accompanied by several subsections, describing, in detail, the materials synthesis, and the electrodes/devices fabrication. The results obtained through all used techniques, such as FESEM, ATR-FTIR, XRD, BET, BJH, CV, GCD and EIS spectroscopy, were discussed both for materials and supercapacitor devices. 

The optimized f-CNP electrodes, functionalized with 5 wt% PEDOT, exhibited very high capacitive properties, showing an extraordinary specific capacitance of 258.7 F/g, at a current density of 0.25 A/g.

## 2. Materials and Methods

Alfa Aesar, Waltham, MA, USA, provided the monomer, 3,4-ethylenedioxythiopehene, EDOT (97%), and the oxidizing agent, ammonium peroxydisulfate, APS (98%). The waste onion peels were used as precursors for the preparation of carbon nanoparticles and were obtained from the local vegetable market. Finar Limited, India has supplied AR grade HNO_3_ and H_2_SO_4_ for acid functionalization/activation of carbon compounds. Polyvinylidene difluoride (PVDF) was supplied by SIGMA ALDRICH while AR grade N-methyl-2-pyrrolidone (NMP) and KOH were provided from SD Fine chemicals. The de-ionized water utilized in this study had a resistivity of 18.2 M-cm. Ethanol (Merk, Bengaluru, India) and all reagents were used without additional purification as received. 

### 2.1. Materials Synthesis

#### 2.1.1. Preparation of Poly(3,4-Ethylenedioxythiophene)

In-situ oxidative polymerization was used to synthesize poly(3,4-ethelyenedioxythiophene). In a typical experiment, an aqueous monomer solution was placed in a three-neck round bottom flask having 100 mL of de-ionized water and magnetically agitated for one hour. The addition of APS as an oxidizing agent to the aforementioned solution in a 1:2 molar ratio (EDOT:APS) initiated the chemical oxidative polymerization of EDOT. At room temperature, the polymerization reaction was allowed to continue for 20 h. To eliminate the residues, the resultant dark blue color sample was washed with a large amount of an ethanol-deionized water mixture and dried for 24 h at 60 °C.

#### 2.1.2. Preparation of Functionalized Carbon Nanoparticles (f-CNPs) and PEDOT-Doped Functionalized Carbon Nanoparticles (f-CNP-PEDOT) Nanocomposites

Functionalized carbon nanoparticles were prepared from waste onion peels. The waste onion peels were ground to a fine powder. The powder was sieved in a 60 µm mesh and was used as a precursor to carbon nanoparticles (CNPs). The CNPs were prepared by pyrolyzing the precursor at 1000 °C in the nitrogenated atmosphere for one hour. A more detailed description of the synthesis of such CNPs is described in our earlier works [18,19,20,21,22,23]. The obtained CNPs were further subjected to sonication in an acidic mixture of HNO_3_:H_2_SO_4_ (1:3) for 3 h. The mixture was filtered and washed with Millipore water until it reached a pH of 7, i.e., neutral. The obtained particles were dried overnight and were labelled f-CNPs (i.e., functionalized carbon nanoparticles). These f-CNPs were doped with in-house synthesized PEDOT by mixing f-CNPs and PEDOT in ethanol using a magnetic stirrer for 36 h at 500 rpm. The leftover ethanol was evaporated at room temperature. The carbon-PEDOT residue was dried overnight at 80 °C and labelled as f-CNP-PEDOT. Five f-CNP-PEDOT nanocomposites were prepared and labelled as f-CNP-PEDOT1, f-CNP-PEDOT2.5, f-CNP-PEDOT5, f-CNP-PEDOT10 and f-CNP-PEDOT20, where 1, 2.5, 5, 10 and 20 represent the weight percentage of PEDOT.

### 2.2. Electrodes Fabrication

**Figure 1:**
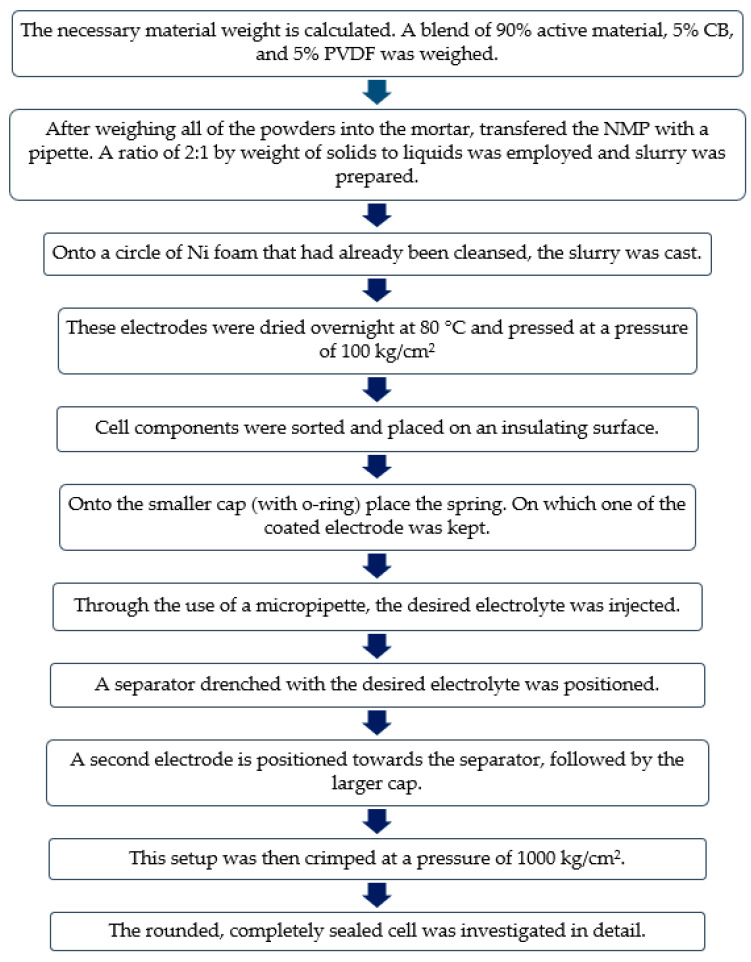
Flow chart of cell devices fabrication.

f-CNP-PEDOT working electrodes were made by applying a homogenized slurry of active material, carbon black and polyvinylidene difluoride (PVDF) [in 80%:10%:10% ratio, respectively] prepared using a solvent, i.e., N-methyl-2-pyrrolidone, on 1 cm × 1 cm pre-cleaned nickel foam substrate. These were then dried overnight at 80 °C and pressed with a mass loading of ~4 mg at a pressure of 100 kg/cm^2^. A three-electrode system was used to conduct electrochemical experiments in 1 M KOH electrolyte. The auxiliary electrode was platinum wire, while the reference electrode was a saturated calomel electrode. Using f-CNP-PEDOT5 as the active material and an identical mass loading (~2.5 mg) on the cathode and anode, a symmetric supercapacitor of the CR2032-type coin cell was made. They were separated using 1 M KOH electrolyte and Whatman^®^ glass microfiber filters (GF/D). The flow chart for the cell fabrication is provided below in Figure 1:

### 2.3. Characterization Thechniques

Attenuated transmittance spectra of f-CNPs and pristine PEDOT, and f-CNP-PEDOT nanocomposites were obtained in the wave number range of 500 to 3500 cm^−1^ using Bruker Alpha FTIR spectrometer. The XRD patterns of the samples were collected by the Bruker D8 advanced X-ray diffractometer in the angular range of 10° to 60° using Cu-Kα radiation (*λ* = 1.5406 Å) as the excitation source. A Carl Zeiss Ultra55 (Oberkochen, Germany) field-emission scanning electron microscope (FESEM) was used to capture the morphology of the samples. The energy dispersive X-ray spectroscopy (EDX) data were obtained using Oxford INCA x-sight X-ray detector (Oxford Instruments, Abingdon, UK) attached with Ultra 55 instruments.

An electrochemical workstation (AUTOLAB M204, Utrecht, The Netherlands) was used to conduct the electrochemical tests. In the potential range of 0 to −1 V, the cyclic voltametric (CV) studies for three-electrode systems were performed at varied scan rates ranging from 10 mV/s to 100 mV/s. At current densities ranging from 0.1 A/g to 5 A/g, the constant current galvanostatic charge discharge tests (GCD) were performed. In the potential range of 0 to 1.4 V, CV analysis was carried out for a CR2032-type symmetric supercapacitor cell. Scanning speeds rates from 5 to 100 mV/s were employed. GCD tests were performed at various current densities (0.1–5 A/g) and in various potential windows (0–1.4 V). The gravimetric specific capacitance, *C*, of a single electrode was computed using the Equation (1) for this two-electrode setup [24].

(1) $$C=\frac{4I\times \Delta t}{m\times \Delta V}$$

where the symbols *I*, Δ*t*, *m*, and Δ*V* indicate the discharge current (A), the discharge time (s), the total loading of active material (g), and the voltage difference (*V*) within Δ*t*, respectively. For the three-electrode system, gravimetric specific capacitance (*C*) was calculated from Equation (2):

(2) $$C=\frac{I\times \Delta t}{m\times \Delta V}$$

where the notations *I*, Δ*t*, *m*, and Δ*V* have the same meaning as in the Equation (1). The electrochemical impedance of the system was measured at the open circuit potential (OCP) in the frequency range from 0.01 Hz to 100 kHz at an AC amplitude of 10 mV. The frequency response analysis of real, *C*′, and imaginary, *C*″, capacitances of a complex capacitance, i.e., C* (= *C*’ – *jC*″), were computed using the equations presented in our previous work [25]. Equations (3) and (4) were used to calculate the energy density, *E*, and power density, *P*, respectively [26,27].

(3) $$E=\frac{C\Delta V^{2}}{7.2}$$

(4) $$P=\frac{E\times 3600}{\Delta t}$$

## 3. Results and Discussion

### 3.1. Attenuated Total Reflectance-Fourier Transform Infrared Spectroscopy (ATR-FTIR)

**Figure 2:**
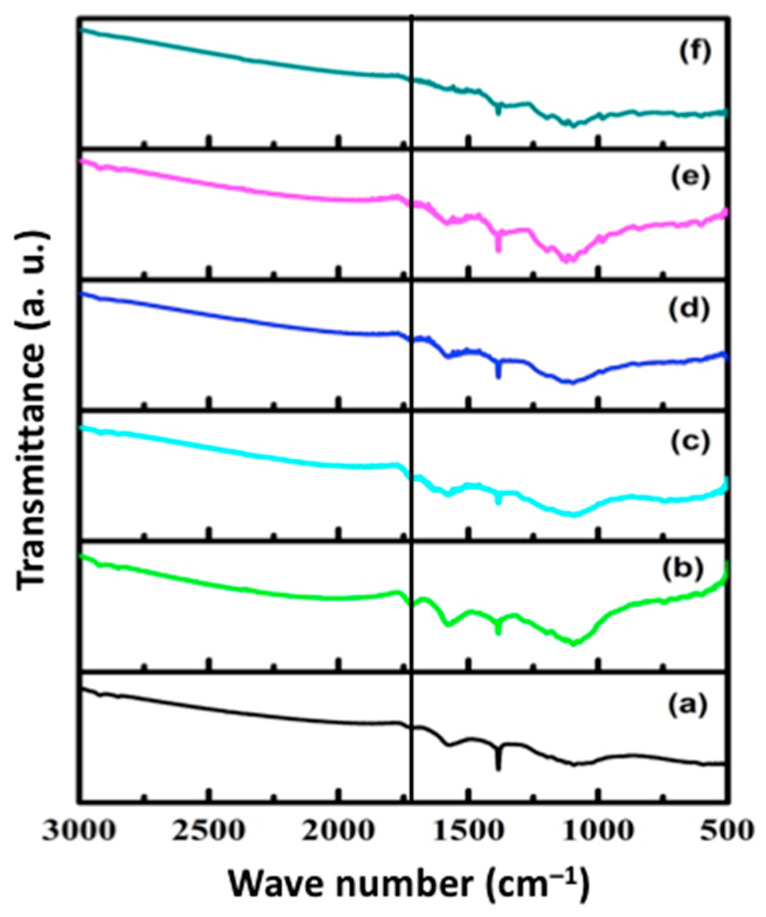
ATR-FTIR spectra of pristine f-CNPs and f-CNP-PEDOT nanocomposites. (**a**) pristine f-CNPs, (**b**) f-CNP-PEDOT1, (**c**) f-CNP-PEDOT2.5, (**d**) f-CNP-PEDOT5, (**e**) f-CNP-PEDOT10, and (**f**) f-CNP-PEDOT20.

ATR-FTIR studies were performed to analyze the functional groups present in the samples. FTIR plots of pristine f-CNPs and f-CNP-PEDOT nanocomposites with different concentrations of PEDOT (1, 2.5, 5, 10, and 20 wt%) are presented in Figure 2. IR spectrum of pristine f-CNPs, Figure 2a, is characterized by a peak at approximately 1092 cm^−1^ which is due to C–O stretching vibration [28]. A peak at 1383 cm^−1^ hints the presence of S=O stretching mode and a peak observed at approximately 1725 cm^−1^ originates due to C=O stretching vibrations [29]. A peak obtained at 1574 cm^−1^, indicated by a straight, corresponds to the carboxylic group stretching vibration. Carboxyl groups play a vital part in the formation of spherical structures and also they provide the possibility of loading additional molecules or ions or functional groups [30].

All these characteristic peaks of pure f-CNPs are evident in the spectra of f-CNP-PEDOT nanocomposites, Figure 2b–f, with a slight shift in some peaks. An un-obvious identification for peaks of filler PEDOT in the f-CNPs-PEDOT nanocomposites is probably due to their information overlapping with that of f-CNPs or being too weak compared to that of f-CNP matrix [14]. 

### 3.2. X-ray Diffraction (XRD) Analysis

**Figure 3:**
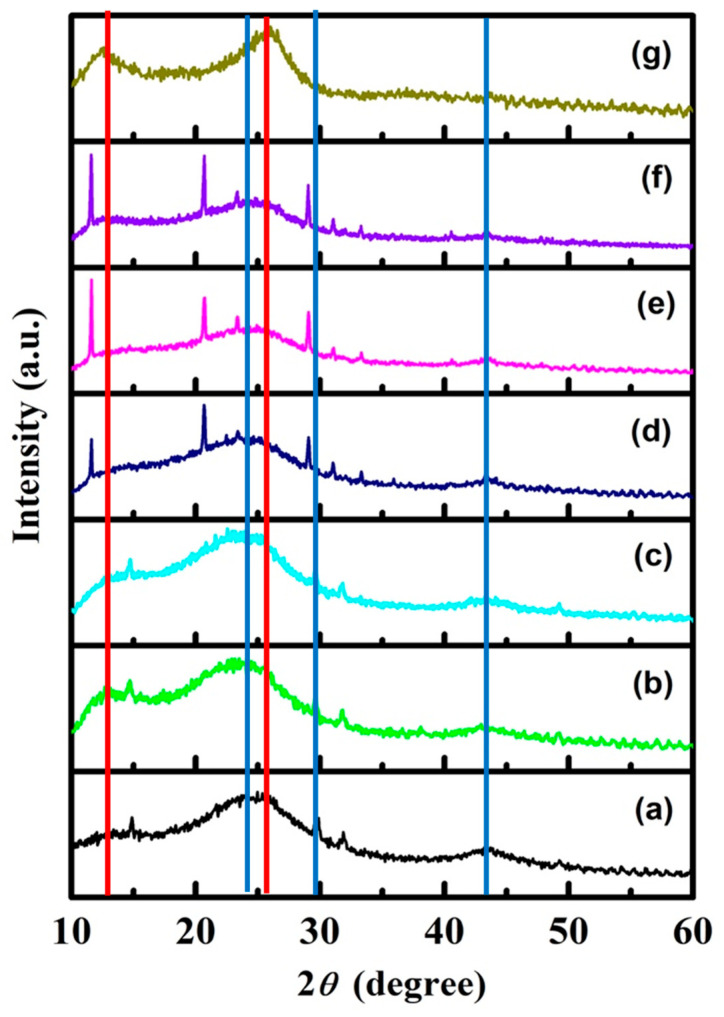
XRD patterns of pristine f-CNPs and f-CNP-PEDOT nanocomposites. (**a**) pristine f-CNPs, (**b**) f-CNP-PEDOT1, (**c**) f-CNP-PEDOT2.5, (**d**) f-CNP-PEDOT5, (**e**) f-CNP-PEDOT10, (**f**) f-CNP-PEDOT20, and (**g**) pure PEDOT. The blue straight lines mark the three XRD peaks at 2*θ* = 24°, 29.3° and 43.5°, whereas the red ones indicate the two PEDOT features.

XRD studies help in the identification of the phase and nature of the synthesized samples. XRD patterns of all samples, f-CNPs, f-CNP-PEDOT nanocomposites, with different concentrations of PEDOT (1, 2.5, 5, 10, and 20 wt%), and pure PEDOT, are presented in Figure 3.

In the XRD pattern of pristine f-CNPs, Figure 2a, a broad peak was observed at 2*θ* = 24° attributed to carbon. In addition, two more weak and narrow peaks obtained at 29.3° and 43.5° correspond to (0 0 2) and (0 0 1) C planes, respectively. A peak observed at 31.7° is maybe due to the presence of calcium oxides or carbonate traces [28,31]. All of these XRD peaks were present in the spectra of Figure 3b–f, whereas the XRD pattern of pure PEDOT (Figure 3g) indicates two main peak at 25.4° (*d* = 3.5 Å), related to the interchain planar ring-stacking distance in PEDOT [32], and at approximately 12.5° due to the π-stacking [33]. 

XRD patterns of f-CNP-PEDOT nanocomposites also show a structural arrangement of carbon atoms. The peaks corresponding to f-CNPs were shifted towards a slightly higher angle at a higher concentration of PEDOT indicating the successful interaction between f-CNPs and PEDOT. Further, as the concentration of PEDOT was increased to 5 wt% a new peak corresponding to PEDOT was observed at approximately 11.7°. This peak has become sharp and more intense with a further increase in the concentration of PEDOT up to 20 wt%. This may be due to the coverage of PEDOT on the porous structure of f-CNPs and the dense structure of f-CNP-PEDOT nanocomposites at higher concentrations.

### 3.3. Field-Emission Scanning Electron Microscopy (FESEM) Analysis

**Figure 4:**
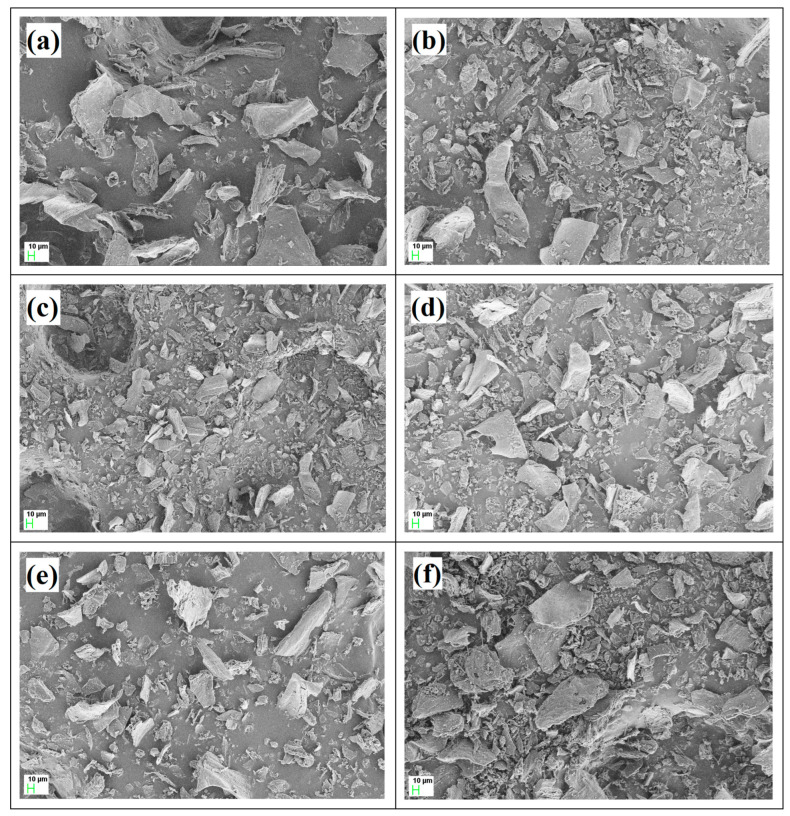
FESEM images of pristine f-CNPs and f-CNP-PEDOT nanocomposites. (**a**) pristine f-CNPs (1.0 KX), (**b**) f-CNP-PEDOT1 (1.0 KX), (**c**) f-CNP-PEDOT2.5 (1.0 KX), (**d**) f-CNP-PEDOT5 (1.0 KX), (**e**) f-CNP-PEDOT10 (1.0 KX), and (**f**) f-CNP-PEDOT20 (1.0 KX).

The morphology of all samples was studied using FESEM. Figure 4a shows the microstructure of pure f-CNPs which indicates the porous morphology. Figure 4b–f show the FESEM micrographs of f-CNP-PEDOT nanocomposites. As the concentration of PEDOT was increased, the dense and compact nature was observed which may be due to the interconnection of f-CNPs with the PEDOT polymer chain. The porous structure facilitates the rapid ion transfer of electrolyte ions. Although a clear distinction between the various samples caused by the insertion of PEDOT at various concentrations is difficult from the analysis of FESEM images alone, it seems that at higher concentrations, the PEDOT might have occupied the pores of f-CNPs resulting in a more dense morphology of nanocomposites. 

The chemical constituents of the samples were examined by EDX and the results are depicted in Table 1. It can be observed that the samples contain the highest amounts of carbon and a significant amount of oxygen. Sulphur is another element, which is present in small quantities in the nanocomposites.

### 3.4. Porosity

**Figure 5:**
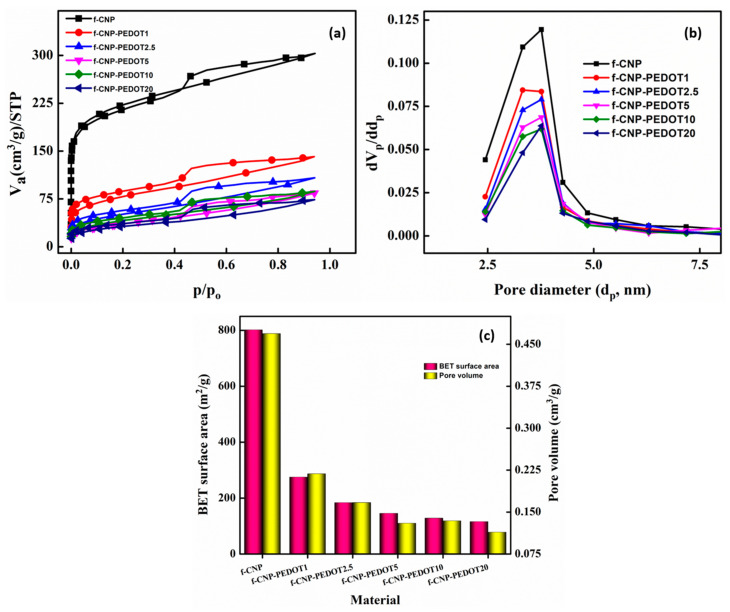
N_2_ adsorption/desorption isotherms (**a**), pore size distribution (**b**), BET surface area and pore volume (**c**) in f-CNP and f-CNP-PEDOT doped (1, 2.5, 5, 10, and 20 wt%) samples.

N_2_ adsorption/desorption isotherms helped in understanding the porous nature of pristine f-CNP and f-CNP-PEDOT nanocomposites. Adsorption isotherms are depicted in graphical form in Figure 5a, with the amount adsorbed plotted versus the equilibrium relative pressure, *p*/*p*_o_. All the samples display-type IV isotherms which are given by mesoporous materials. The isotherms belong to type IVa, where capillary condensation is accompanied by hysteresis. Hysteresis resembles the H4 type. The more pronounced uptake at low *p*/*p*_0_ indicates micropore filling. BET surface area, pore diameter and pore volume are provided in Table S1. As expected, f-CNP has the largest surface area (801.6 m^2^/g) and f-CNP-PEDOT20 has the lowest (116 m^2^/g). Pore size was calculated from the BJH method (Figure 5b). All samples confirmed the presence of mesopores, ranging from size small as 2 nm to larger dimeter of 50 nm. Accordingly, the pore volume also follows the same trend as that of BET surface area (Figure 5c). 

The nitrogen, N_2_, adsorption/desorption results suggest the formation of f-CNP-PEDOT nanocomposites. f-CNP being a highly mesoporous material acts like a porous matrix where PEDOT is deposited. This is supported by the surface area values of samples. The surface area of the nanocomposites decreases with an increase in the concentration of PEDOT from 1 wt% to 20 wt%. One possibility could be, that PEDOT may have occupied the pores in f-CNP, resulting in a reduction in surface area. During the N_2_ sorption experiment, when PEDOTs occupied the pores in f-CNP, the volume and space for N_2_ adsorption decreased, mirroring a reduced surface area for f-CNP-PEDOT samples.

### 3.5. Electrochemical Analysis

#### 3.5.1. Three-Electrode Setup

**Figure 6:**
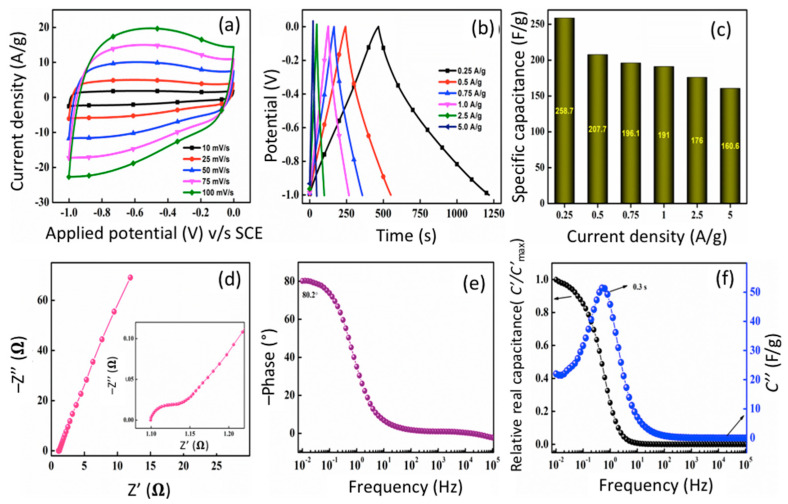
CV of f-CNP-PEDOT5 nanocomposite at different scan rates (**a**), galvanostatic charge discharge (GCD) curves at various current densities (**b**), plot of specific capacitance as a function of current density (**c**), Nyquist impedance plot (inset depicts high-frequency region) (**d**), Bode phase angle plot as a function of frequency (**e**), plot of relative real, *C*′/*C*′_max_, and imaginary, *C*″, part of capacitances with frequency (**f**).

Electrochemical analysis was recorded in three-electrode setup with a 1 M KOH electrolyte. Cyclic voltammetry (CV) curves provide a vital clue regarding the charge storage mechanism. The absence of redox peaks, for f-CNP-PEDOT electrodes, suggests domination of capacitive-type charge storage. The CV curves recorded for pristine f-CNPs, pure PEDOT, f-CNP-PEDOT1, f-CNP-PEDOT2.5, f-CNP-PEDOT5, f-CNP-PEDOT10, and f-CNP-PEDOT20 are compared and provided in Figure S1. No redox peaks were observed for any of the electrodes indicating the domination of capacitive charge storage. CV curves, provided in Figure 6a at various scan rates, suggest higher charge storage in f-CNP-PEDOT5 nanocomposite, compared to curves of Figure S1. The current response increased with an increase in scan rates from 10 mV/s to 100 mV/s. However, this caused slight deformation of the rectangular profile at a higher scan rate which is usually observed in carbon-based electrodes [34,35]. The magnitude of charge storage is indicated by specific capacitance calculated from charge–discharge tests. GCD profile of all the electrodes is provided in Figure S2. All the electrodes showed a linear, symmetrical and triangular response further cementing the EDLC nature of f-CNP and f-CNP-PEDOT nanocomposites. A maximum specific capacitance of 145.7, 54.3, 206.3, 212.4, 258.7, 187.6, and 162.2 F/g was calculated, by Equation (2) for f-CNPs, PEDOT, f-CNP-PEDOT1, f-CNP-PEDOT2.5, f-CNP-PEDOT5, f-CNP-PEDOT10, and f-CNP-PEDOT20 electrodes, respectively, at 0.25 A/g. The GCD profile for f-CNP-PEDOT5 at different current densities is exhibited in Figure 6b and it shows a maximum specific capacitance of 258.7 F/g at 0.25 A/g which was higher than that of f-CNPs and PEDOT indicating a synergetic effect of doping. Specific capacitance as a function of current densities is provided in Figure 6c. Approximately 63% of capacitance was retained when the current densities was increased from 0.25 to 5.0 A/g. 

Electrochemical impedance spectroscopic (EIS) analysis further provided insights into the electrochemical behavior of f-CNP-PEDOT5. Nyquist impedance plots are presented using imaginary, *Z*″, and real, *Z*′, impedances, at various frequencies. The distinctive nature of electrode materials can be assessed for different frequency domains. The Nyquist plot, provided in Figure 6d, for f-CNP-PEDOT5, shows a semicircle at a high-frequency region (inset graph in Figure 6d) followed by a vertical tail. The equivalent series resistance (ESR), *R*_s_ (=1.1 Ω), which is the total internal resistance of the cell was found from the point of intersection at the x-axis at highest applied frequency in the Nyquist plot. A very low charge transfer resistance, *R*_CT_ (=0.05 Ω), was observed indicated by a small semicircle at a high-frequency region. This further confirms the EDLC nature of f-CNP-PEDOT5 nanocomposite indicating physical charge storage, i.e., electrostatic mechanism, and hence charge transfer resistance, *R*_CT_, is negligible. An immediate 45° line from *Rs* was detected at high frequencies, followed by a vertical tail, indicating easy access to active sites on the electrode in a short time, indicating capacitive like behavior. Bode phase angle plot is another way to express impedance nature. f-CNP-PEDOT5 nanocomposite shows a phase angle of −80.2° (Figure 6e) which is close to an ideal capacitor (=−90°). A constant phase angle indicated by a flat line in the high-frequency region can be related to the diffusive resistance of the system. The fast charge movement within the electrode-electrolyte interface can be seen from the absence of a peak at the high-frequency region, and the phase angle shifts to a higher value at a lower frequency. This also means the high charge storage properties of f-CNP-PEDOT5.

Real, *C*′, and imaginary, *C*″, capacitances were calculated and plotted against frequency (Figure 6f). A transition between purely resistive behavior from *C*′ = 0 to *C*′ = 1 is plotted against frequency. For ideal capacitors, the capacitance remains constant as a function of frequency once this transition occurs [36]. f-CNP-PEDOT5 nearly exhibits this behavior by reaching towards saturation. The variation in imaginary capacitance (*C*″) with frequency is shown in Figure 6f. The transition from pure resistive to pure capacitive is indicated by the peak of the curve. The frequency at which this shift occurs, *f*_0_, was observed to shift towards higher frequencies. The reciprocal of this characteristic frequency (1/2π*f*_0_) yields a time constant, *τ*, which is a quantitative measure of how quickly the cell can be charged and discharged reversibly [36]. The computed time constant of 0.3 s indicates that f-CNP-PEDOT5 has a good EDLC nature.

The porous nature of the active material plays a critical role in obtaining a high capacitance in the carbon-based electrodes for supercapacitors. Highly mesoporous f-CNPs have been used as a carbon matrix for accumulating PEDOT in their pores, as evidenced by porosity results. When f-CNP-PEDOT nanocomposites were formed, it appears that a synergetic effect predominated, resulting in enhanced capacitance. f-CNP with a higher surface area can accumulate more ions and charges from the electrolyte and PEDOT (with its polymeric chain consisting of electronegative atoms like O and S) could further increase ion accumulation, and thus charge [37]. However, as the amount of PEDOT doped in f-CNPs increased from 1 to 20 wt%, more pores in the f-CNPs may have been occupied by PEDOT, resulting in channel and pores blockage which inhibits easy ion flow from electrolytes. This diminishes the active material’s capacity to store charges, resulting in a lower capacitance value. A decrease in surface area and pore volume was observed with an increase in the PEDOT doping weight percentage beyond 5 wt%, indicating more adsorption of PEDOT onto the f-CNP matrix. The synergetic effect of PEDOT and f-CNP appears to disappear due to a decrease in surface area in f-CNP-PEDOT nanocomposites (from 801.6 to 116 m^2^/g). The 5 wt% doping of PEDOT onto the f-CNP matrix appears to elicit a better electrochemical response than the others, implying that the dopant should be limited to 5 wt%. Hence, f-CNP-PEDOT5 nanocomposite was used as an active material for the fabrication of a symmetric aqueous supercapacitor device.

#### 3.5.2. Aqueous Symmetric Supercapacitor

**Figure 7:**
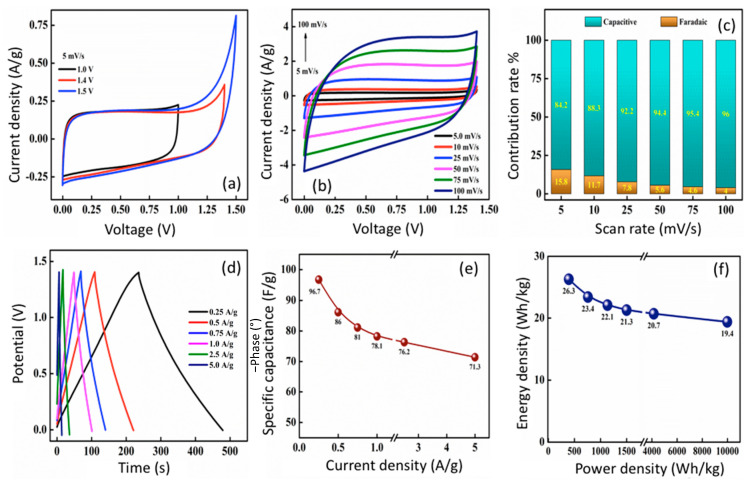
Cyclic voltammetry (CV) of f-CP5 at the different voltages at 5 mV/s (**a**) and different scan rates under 1.4 V (**b**), capacitive and faradaic charge storage contribution of f-CP5 at various scan rates (**c**), galvanostatic charge discharge (CGD) curves at different current densities operated at 1.4 V (**d**), specific capacitance as a function of current densities (**e**), and Ragone plot of f-CP5 (**f**).

A symmetric supercapacitor of CR2032-type aqueous cell was fabricated using f-CNP-PEDOT5 nanocomposite as the active material for the cathode and anode, and 1 M KOH as the electrolyte. This will be referred to as f-CP5 from now on. Figure S3a–c shows the CV at various scan rates operated under 1, 1.4, and 1.5 V. CV curves exhibit a nearly rectangular profile at lower scan rates and a skewed quasi rectangular shape at higher scan rates. CV curves at different voltages were compared at a scan rate of 5 mV/s (Figure 7a). The overvoltage was observed at 1.5 V, and thus further studies were conducted where the cell was operated at 1.4 V. The CV profile at various scan rates (5 to 100 mV/s) shows the domination of EDLC nature (Figure 7b). Equation (5) was utilized to mathematically quantify the type of charge storage mechanism [26],

(5) logi=loga+blogv

where current response, *i*, is related to sweep rate, ν, by the sum of capacitive and diffusion processes, and ‘*a*’ and ‘*b*’ are the adjustable parameters. The linear response of log *i* versus log *v* (Figure S3d) fits well (*R*^2^ = 0.99) with the linear equation (*y* = *a* + *b* · *x*), and the slope gives the value of ‘*b’* = 0.98, which is closer to unity. This shows that the dominance of the capacitive mechanism could be attributable to a linear combination of both diffusion and capacitive effects [38]. However, this method does not allow for the quantification of the individual mechanism. As a result, using Equation (6), the CV response was further examined in order to identify individual contributions,

(6) $$\frac{i(V)}{v^{1/2}}=k_{1}v^{1/2}+k_{2}$$

where *k*_1_*ν* is a measure of capacitive response and *k*_2_*ν*^1/2^ is a measure of a diffusion-controlled process [39]. The slope and intercept obtained from the linear plot of $\frac{i(V)}{v^{1/2}}\mathrm{vs}\;k_{1}v^{1/2}$ (Figure S3e) were used to calculate *k*_1_ and *k*_2_. The linear fit has *R*^2^ of 0.98, indicating that it is reliable. The contribution ratio as a function of scan rate was calculated using the obtained *k*_1_ and *k*_2_ values (Figure 7c). As can be observed from the graph, the diffusion-type (faradaic) current response contributes 15.8% of the total current response at the lowest scan rate, i.e., 5 mV/s. As the scan rate increased, it decreased to ~4%. The current response was primarily caused by capacitive-type charge storage, regardless of the scan rate.

The GCD tests were carried out at the maximum operating voltage of the cell, i.e., 1.4 V, and the results are displayed in Figure 7d. The symmetric, triangular, and linear-shaped curves complement the results of CV analysis. The experiments were carried out at current densities ranging from 0.1 to 5.0 A/g. The gravimetric specific capacitance of f-CP5 was further calculated from GCD curves at various current densities. Figure 7e shows the decrease in capacitance with an increase in current density from 96.7 F/g (at 0.25 A/g) to 71.3 F/g (at 5.0 A/g). Even at the higher current density, approximately 74% of the initial capacitance was retained, promising the commercial viability of the device. The energy density and power density of the cell were determined using Equations (3) and (4), and is presented in Ragone plot, i.e., Figure 7f. The device had a maximum energy density of 26.3 Wh/kg (at 391.6 W/kg) and retained nearly 74% of this energy density (=19.4 Wh/kg) at a high-power density of 9982 W/kg, indicating that the f-CNP-PEDOT5 nanocomposite has a wide range of potential applications. Approximately 86% of capacitance retention by the end of 10,000 cycles is very promising for commercial applications. 

**Figure 8:**
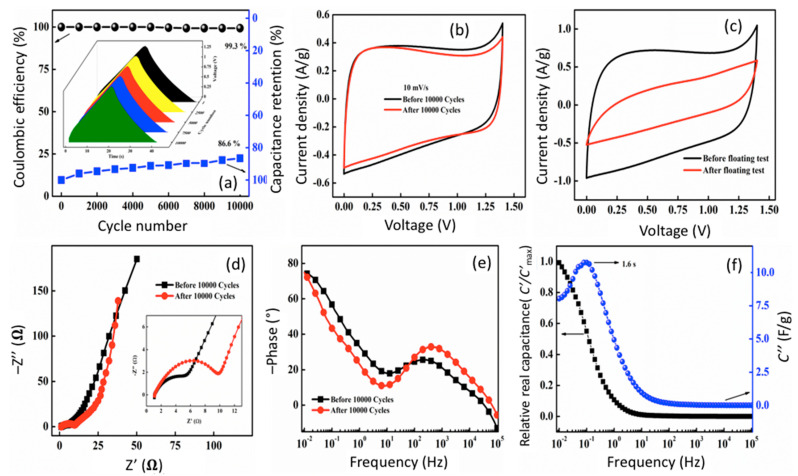
Coulombic efficiency and capacitance retention over 10,000 GCD cycles (inset shows the charge discharge curves at regular intervals over 10,000 cycles) (**a**), comparison of CV profile of f-CNP-PEDOT5 device before and after 10,000 GCD cycles at 10 mV/s (**b**) and floating test (**c**), Nyquist impedance plot before and after 10,000 charge discharge cycles (inset shows Nyquist impedance plot at high-frequency region) (**d**), Bode phase angle plot before and after 10,000 GCD cycles (**e**), and plot of relative real, *C*′/*C*′_max_, and imaginary, *C*″, part capacitances with frequency (**f**).

The stability of the f-CNP-PEDOT5 device was assessed over 10,000 charge/discharge cycles carried out at a constant current density of 2 A/g operated at 1.4 V. During 10,000 cycles, the device displayed an outstanding coulombic efficiency of 99.3% (Figure 8a). The cell also showed superior capacitance retention of 86.6% after 10,000 cycles, indicating that the electrode-electrolyte assembly is stable. The charge/discharge curves of f-CNP-PEDOT5 device at regular cycling intervals during 10,000 charge–discharge cycles are shown in the inset graph of Figure 8a. The uniformity of the curves at different cycle numbers proves the stability and the possibility of the commercial viability of the f-CNP-PEDOT5. The device was further tested for its possible loss in current response through CV curves. Figure 8b shows CV curves recorded before and after 10,000 charge discharge cycles. Even after 10,000 charge discharge cycles, the cell retained 93% of the current response. The floating method (Aging) was used to test the stability of the device. f-CP5-PEDOT5 was subjected to charging at a constant current (1.0 A/g) at a maximum operating voltage (1.4 V) for 100 h. The CV was measured after 100 h and compared with the CV profile of f-CP5, before starting the floating test (Figure 8c). Before and after 10,000 charge/discharge cycles, the impedance data was recorded (Figure 8d). The fitted values are provided in Table 2. In the high-frequency region, we observed a very small inductance which increased after cycles, likely due to structural changes in the circuit and electrode materials. The solution resistance *R_s_* increased by about 3.4%, from 1.045 Ω to 1.081 Ω. This small increase may be due to minor changes in the electrolyte composition or electrode-electrolyte interface. The charge transfer resistance *R*_CT_ nearly doubled, increasing by 81% from 5.435 Ω to 9.848 Ω. This large rise indicates increased difficulty in charge transfer at the electrode surface, likely due to surface film modification or other changes during cycling [40]. The diffusion-related constant phase element, Ss^n^, derived from the curve of Figure 6d, − Z″ vs. Z′, and Figure 6e,—Phase vs. Frequency, increased by 54%, from 0.0608 Ss^n^ to 0.0936 Ss^n^, where the n exponent passes from 0.873 to 0.923, is indicative of the quality of the electrodes material. This suggests changes in the diffusion kinetics, possibly due to altered pore structure or surface chemistry of the carbon electrodes. In fact, the CPE exponent n increased by 0.05 (from 0.873 to 0.923), getting closer to 1. This indicates the system is becoming more capacitive in nature after cycling, consistent with the other parameter changes. Bode plots were used to determine the phase angle before and after 10,000 cycles (Figure 8e). The phase angles of nearly 76° and 74.6° were obtained before and after 10,000 cycles, respectively. The relative real, *C*′/*C*′_max_, and imaginary, *C*″, part of capacitance as a function of frequency are plotted in Figure 8f. This plot provides a convenient method to show the frequency response of supercapacitors because frequency is the dependent variable, unlike Nyquist plots that have frequency buried in both the real and imaginary impedance terms. The graph (*C*′/*C*′_max_ v/s Frequency) shows a transition between purely resistive behavior to capacitive behavior (*C*′/*C*′_max_ = 0 to *C*′/*C*′_max_ = 1). Ideally once this transition happens the capacitance must remain invariant to frequency change in applications. *C*″ *v*/*s* Frequency plot, evolution of imaginary capacitance with frequency is shown. The maximum of the curve is a characteristic of the entire system and can be roughly described as the point where the circuit goes from purely resistive to purely capacitive. The reciprocal of this characteristic frequency (where the maximum appears) yields a time constant, *τ*, i.e., a quantitative measure of how fast the device can be charged and discharged reversibly. The time constant, *τ*, obtained from the maximum of the peak was 1.6 s.

The electrodes material exhibited superior performance compared to earlier published reports (Table 3).

## 4. Conclusions

f-CNP-PEDOT-doped nanocomposites were prepared by varying the concentration of PEDOT (i.e., 1, 2.5, 5, 10, and 20 wt%). ATR-FTIR and XRD studies indicate the successful formation of nanocomposites. The mesoporous nature of the samples was observed by N_2_ adsorption/desorption experiments. The applicability of f-CNP-PEDOT nanocomposites as a supercapacitor material was tested using a three-electrode system. Among the prepared nanocomposites, f-CNP-PEDOT5 nanocomposite exhibited better performance and maximum specific capacitance of 258.7 F/g at 0.25 A/g which is higher than that of f-CNP and PEDOT signifying a synergetic effect of doping. Porosity studies helped in predicting the reason for the decrease in capacitance with an increase in PEDOT doping percentage onto f-CNPs. Symmetric aqueous supercapacitor cells were fabricated with f-CNP-PEDOT5 nanocomposite as the active material. The cell showed a high capacitance of 96.7 F/g at 1.4 V. The synergetic effect seems to have helped in widening the potential window from 1 to 1.4 V when an aqueous electrolyte was used. 10,000 charge–discharge cycles at 2 A/g had minimal effect on the capacity of f-CNP-PEDOT5 nanocomposite to hold charges. Our symmetric aqueous supercapacitor cells exhibited higher performances compared to previous works reported in literature. These new results indicate the potential of f-CNP-PEDOT5 nanocomposite as electrode material in practical devices/cells. 

## Figures and Tables

**Table 1:** Elemental composition of pure f-CNPs and f-CNP-PEDOT nanocomposites.

Samples	Elemental Composition (%)		
	C	O	S
Pristine f-CNP	89.78	10.22	-
f-CNP-PEDOT1	84.84	15.16	-
f-CNP-PEDOT2.5	83.89	15.95	0.16
f-CNP-PEDOT5	81.71	17.66	0.63
f-CNP-PEDOT10	88.33	11.44	0.23
f-CNP-PEDOT20	81.96	17.24	0.80

**Table 2:** Circuit parameters of f-CNP-PEDOT5 device before and after 10,000 charge-discharge cycles.

Circuit Parameters	Values	
	Before 10,000 Cycles	After 10,000 Cycles
Inductance	7.51 × 10^−9^ H	2.816 × 10^−7^ H
Solution resistance (*Rs*)	1.045 Ω	1.081 Ω
Charge transfer resistance (*R*_CT_)	5.435 Ω	9.848 Ω
Constant phase element (Diffusion)	0.0608 Ss^n^	0.0936 Ss^n^
n	0.873	0.923

**Table 3:** Comparison of circuit parameters obtained in the present work with some of earlier published research.

Biomass Precursor	Potential Window (V)	Electrolyte	Energy Density (Wh/kg)	Efficiency Retainment (%)	Capacitance Retention (%)	Reference
Quinoa	1.0	6.0 M KOH	9.5	100 @ 10,000 cycles	~93	[41]
IHPC	1.3	Mixed alkaline electrolyte	19.7	89	-	[42]
Platanus bark	3.0	TEABF_4_/ACN	34.6	-	70.8 @ 5000 cycles	[43]
Coconut	1.8	LiBF_4_ in EC/DMC	91.1	89 @ 1000 cycles	99 @ 1000 cycles	[44]
Corn silks	2.7	1.0 M MeEt_3_NBF_4_/PC	16.4	-	81.6 @ 10,000 cycles	[45]
Acacia auriculiformis pods	2.3	1.0 M TEABF_4_/ ACN	16.7	100 @ 10,000 cycles	~93.2 @ 10,000 cycles	[26]
f-CNP-PEDOT	1.4	1.0 M KOH	26.3	99.3 @ 10,000 cycles	86.6 @ 10,000 cycles	Present work

## Data Availability

Data is contained within the article.

## Supplementary Materials

The following supporting information can be downloaded at: https://www.mdpi.com/article/10.3390/nano14141222/s1, Table S1: Porosity data of f-CNP and f-CNP-PEDOT samples; Figure S1: Cyclic voltammetric plots at different sweep rates (10, 25, 50, 75 and 100 mV/s) off-CNP (a), PEDOT (b), f-CNP-PEDOT1 (c), f-CNP-PEDOT2.5 (d), f-CNP-PEDOT10 (e), f-CNP-PEDOT20 (f) against standard saturated calomel electrode; comparative CV profile of all the samples at 25 mV/s sweep rate in 1.0 M KOH (g); Figure S2: GCD profile of f-CNP (a), PEDOT (b), f-CNP-PEDOT1 (c), f-CNP-PEDOT2.5 (d), f-CNP-PEDOT10 (e), f-CNP-PEDOT20 (f) at various current densities, a comparative GCD plot of all the samples at 0.25 A/g (g), specific capacitance from GCD curves for all the samples at 0.25 A/g (h); Figure S3: CV of f-CP5 operated under 1.0 V (a), 1.4 V (b) and 1.5 V (c) at various sweep rates (5 to 100 mV/s); plot of log i v/s log ν to determine the nature of charge storage mechanism (d); measure of capacitive and diffusion-controlled mechanism (e); a comparative GCD plot at 1.4 and 1 V (f).
