# Supplementary material for: PEDOT-Doped Mesoporous Nanocarbon Electrodes for High Capacitive Aqueous Symmetric Supercapacitors
Source: Nanomaterials (Basel). 2024 Jul 18;14(14):1222. doi: 10.3390/nano14141222 (PMC11279981; doi:10.3390/nano14141222)
Supplement: Supplementary file 1 [file nanomaterials-14-01222-s001.zip › nanomaterials-3093673-supplementary.pdf]

PEDOT-Doped Mesoporous Nanocarbon Electrodes for High Capacitive Aqueous Symmetric Supercapacitor

Mohsina Taj et al.,

Supplementary information

Table S1. Porosity data of f-CNP and f-CNP-PEDOT samples.

**Material**	**BET surface area (m^2/g)**	**Pore diameter (nm)**	**Pore volume (cm^3/g)**
f-CNP	801.6	2.3	0.469
f-CNP-PEDOT1	275.3	3.2	0.218
f-CNP-PEDOT2.5	183.7	3.6	0.167
f-CNP-PEDOT5	145.5	3.7	0.134
f-CNP-PEDOT10	128.2	4.1	0.130
f-CNP-PEDOT20	116.0	4.0	0.114

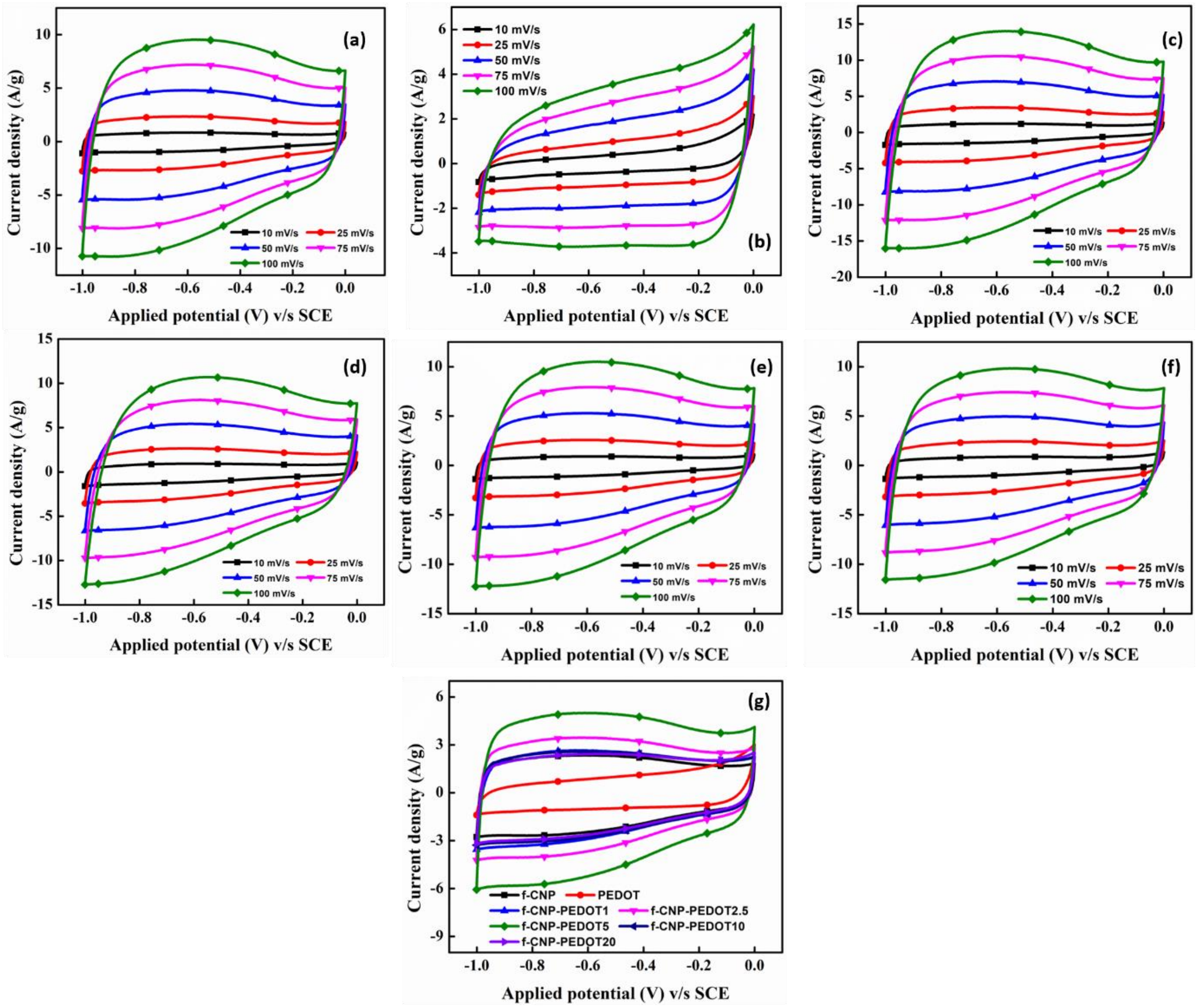

Figure S1. Cyclic voltammetric plots at different sweep rates (10, 25, 50, 75 and 100 mV/s) off-CNP (a), PEDOT (b), f-CNP-PEDOT1 (c), f-CNP-PEDOT2.5 (d), f-CNP-PEDOT10 (e), f-CNP-PEDOT20 (f) against standard saturated calomel electrode; comparative CV profile of all the samples at 25 mV/s sweep rate in 1.0 M KOH (g).

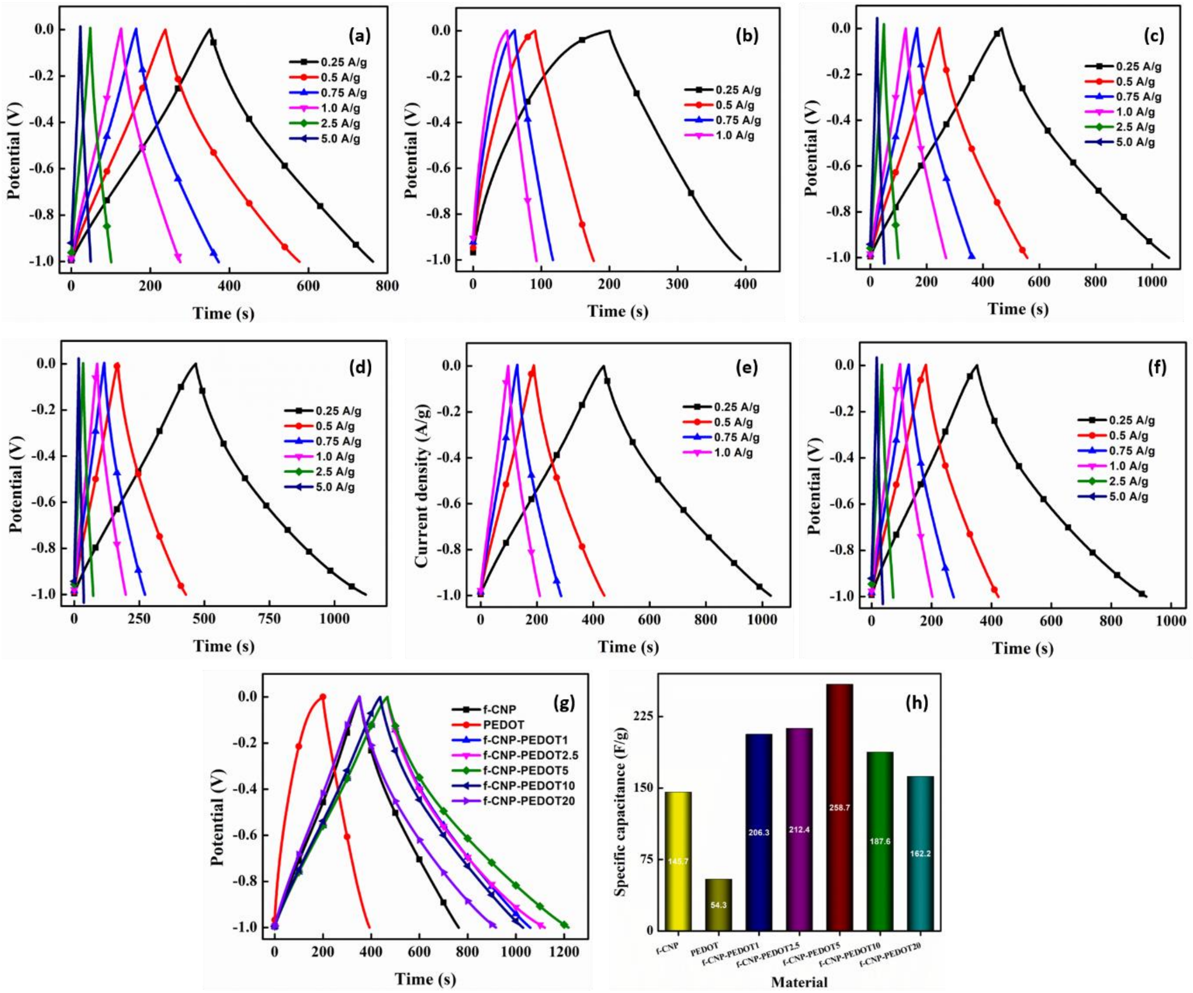

Figure S2. GCD profile of f-CNP (a), PEDOT (b), f-CNP-PEDOT1 (c), f-CNP-PEDOT2.5 (d), f-CNP-PEDOT10 (e), f-CNP-PEDOT20 (f) at various current densities, a comparative GCD plot of all the samples at 0.25 A/g (g), specific capacitance from GCD curves for all the samples at 0.25 A/g (h).

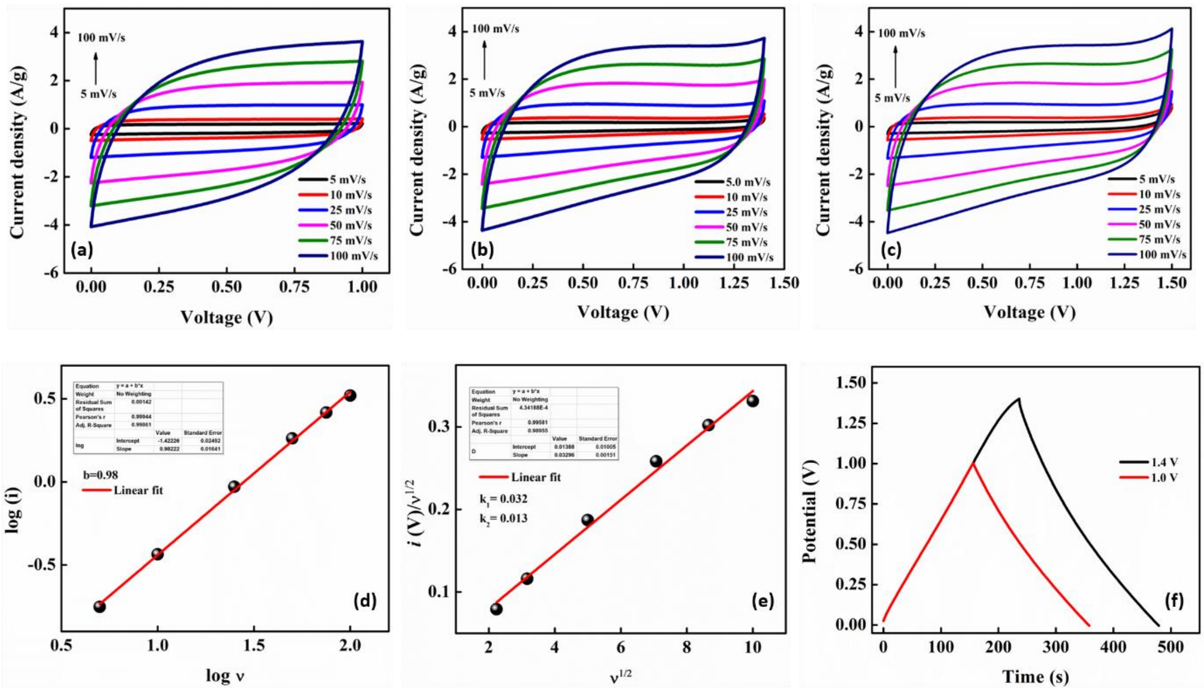

Figure S3. CV of f-CP5 operated under 1.0 V (a), 1.4 V (b) and 1.5 V (c) at various sweep rates (5 to 100 mV/s); plot of log *i* v/s log *ν* to determine the nature of charge storage mechanism (d); measure of capacitive and diffusion-controlled mechanism (e); a comparative GCD plot at 1.4 and 1 V (f).
